# A highly efficient porous rod-like Ce-doped ZnO photocatalyst for the degradation of dye contaminants in water

**DOI:** 10.3762/bjnano.10.115

**Published:** 2019-06-03

**Authors:** Binjing Hu, Qiang Sun, Chengyi Zuo, Yunxin Pei, Siwei Yang, Hui Zheng, Fangming Liu

**Affiliations:** 1 College of Material, Chemistry and Chemical Engineering, Hangzhou Normal University, Hangzhou 311121, P.R. China; 2 Particulate Fluids Processing Centre, Department of Chemical Engineering, The University of Melbourne, Parkville, Victoria 3010, Australia; 3 ARC Centre of Excellence for Nanoscale BioPhotonics (CNBP), School of Science, RMIT University, Melbourne, VIC 3001, Australia

**Keywords:** Ce-doped ZnO, photocatalyst, rhodamine B, solar degradation, surface shape

## Abstract

A mild and simple method was developed to synthesize a highly efficient photocatalyst comprised of Ce-doped ZnO rods and optimal synthesis conditions were determined by testing samples with different Ce/ZnO molar ratios calcined at 500 °C for 3 hours via a one-step pyrolysis method. The photocatalytic activity was assessed by the degradation of a common dye pollutant found in wastewater, rhodamine B (RhB), using a sunlight simulator. The results showed that ZnO doped with 3% Ce exhibits the highest RhB degradation rate. To understand the crystal structure, elemental state, surface morphology and chemical composition, the photocatalysts were characterized by X-ray diffraction (XRD), X-ray photoelectron spectroscopy (XPS), scanning electron microscope (SEM) and inductively coupled plasma emission spectroscopy (ICP), respectively. The newly developed, robust, field-only surface integral method was employed to explore the relationship between the remarkable catalytic effect and the catalyst shape and porous microstructure. The computational results showed that the dipole-like field covers the entire surface of the rod-like Ce-doped ZnO photocatalyst and is present over the entire range of wavelengths considered. The optimum degradation conditions were determined by orthogonal tests and range analysis, including the concentration of RhB and catalyst, pH value and temperature. The results indicate that the pH value is the main influential factor in the photocatalytic degradation process and the optimal experimental conditions to achieve the maximum degradation rate of 97.66% in 2 hours are as follows: concentration (RhB) = 10 mg/L, concentration (catalyst) = 0.7 g/L, pH 9.0 and *T* = 50 °C. These optimum conditions supply a helpful reference for large-scale wastewater degradation containing the common water contaminant RhB.

## Introduction

Organic dyes used in the textile and dye industries account for a large proportion of pollutants in wastewater. Most of the organic dyes used are difficult to degrade, resulting in irreversible damage to the environment [1]. Although many efficient approaches have been applied to manage this problem, including membrane separation, adsorption, coagulation and microbial degradation [2], the disadvantages of these methods are many, such as generation of secondary pollution, refractory degradation products and high costs, all of which have greatly limited their practical application [3–5]. Recently, photocatalytic degradation of organic dyes using semiconductors has attracted much attention [6]. This refers to the process in which organic compounds are gradually oxidized into inorganic compounds or even H_2_O and CO_2_ under the synergistic effects of light and photocatalysis.

ZnO is one of the most suitable catalysts in many industries given its inexpensive, non-toxic, efficient and anti-corrosion properties [7]. Nevertheless, it still has some disadvantages with respect to photocatalysis. For example, it has a narrow response range, low quantum efficiency, and its photogenerated electron–hole pairs are easy to recombine. The photocatalytic performance can be greatly affected by the particle size, morphology and concentration [8–9]. As such, it is possible to modify these ZnO properties to enhance its photocatalytic efficiency. Doping ZnO with rare-earth ions is an attractive strategy to improve its photocatalytic activity by modifying its surface morphology [10–11]. Wang et al. [12] prepared Ce-doped ZnO with different doping levels by using a one-step solution method, using methylene blue as the target pollutant for photodegradation. After exposure to light for 200 minutes, the pure ZnO achieved a degradation rate of 48.36% whereas 1% Ce/ZnO exhibited the best activity among the as-synthesized products (96.11%). It was found that a moderate amount of cerium doping can significantly improve the photocatalytic activity of ZnO. It was hypothesized that when cerium is mixed with ZnO, the electron/hole separation would be facilitated and the ZnO absorption spectrum response range could be expanded due to the modification of the particle size, morphology and concentration, which consequently improves the utilization rate of photons and enhances the photocatalytic activity [13].

In recent years, metal organic frameworks (MOFs) have been intensively investigated and widely utilized in various fields, such as electrocatalysis [14], heterogeneous catalysis [15] and photocatalysis [16]. Yang et al. [17] reported that Ga-MOF displayed moderate to high catalytic activity of decarboxylation. Generally, zinc oxide materials can be synthesized via a hydrothermal process [18], sol–gel method [19], microemulsion method [20], among others. Due to its narrow band, the application of zinc oxide is limited, thus it is common to modify ZnO using rare-earth ions [9]. Koao et al. [21] synthesized pristine and Ce-doped ZnO with various doping concentrations via a chemical bath method. The morphology of the structures turned from flower-like to mixed morphology with pyramid shapes. Chouchene et al. [22] prepared Ce-doped ZnO nanorods by a solvothermal method. However, to the best of our knowledge, the synthesis of rod-like Ce-doped ZnO (abbreviated as CZO [9]) by pyrolysis derived from ZIF-8 (a zeolitic imidazolate framework, ZIF) has not been reported. As one of the most frequently used MOFs, ZIF-8 (2-methylimidazole zinc salt) has potential applications in gas storage, catalysis, etc. [23]. Its merits not only lie in the many facile synthetic methods, but on the good thermal stability and microporous crystalline structure of the material [24]. Also, when treated as a template, ZIF-8 is likely to be converted into a stable and porous material with a large surface area and high photocatalytic efficiency [25]. As such, in this work, we synthesize pure ZnO and CZO derived from ZIF-8. Our results demonstrate that CZO prepared in this way does have a remarkable photocatalytic activity that performs better than those reported previously in the literature [9].

To understand the impressive catalytic effect from the perspective of catalyst shape and porous microstructure, the robust field-only surface integral method was used to explore the possible mechanism of this rod-like Ce-doped ZnO photocatalyst.

## Results and Discussion

### Photocatalytic activity of CZO

**Figure 1:**
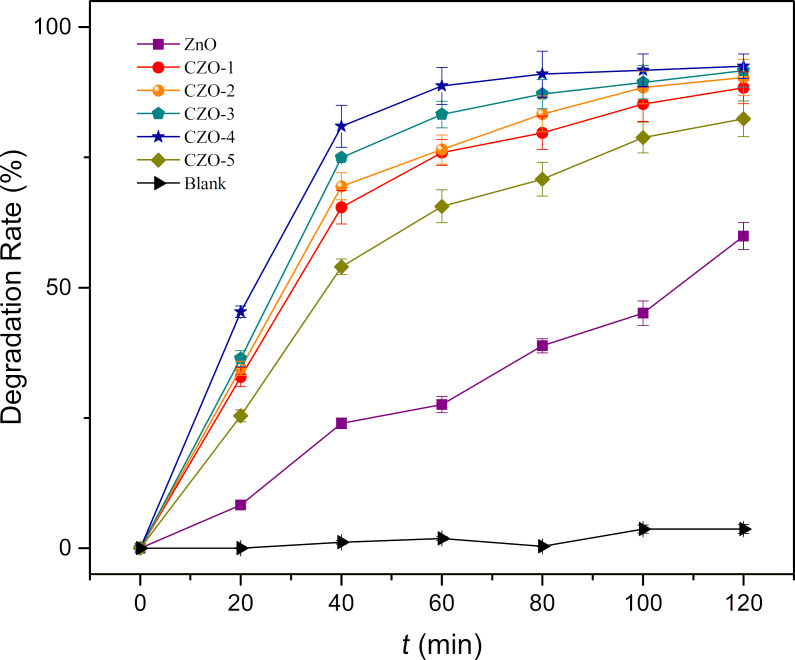
Photocatalytic activity of samples with different Ce doping percentages.

The effect of different Ce/ZnO molar ratios on the photocatalytic activity of ZnO is summarized in Figure 1. The degradation rates of RhB in 2 h were calculated as 59.91%, 88.34%, 90.34%, 91.67%, 92.48% and 82.4% for ZnO, CZO-1, CZO-2, CZO-3, CZO-4 and CZO-5, respectively, corresponding to samples with a Ce concentration of 0.5%, 1%, 2%, 3%, and 4%, respectively.

The experimental results demonstrated that all CZO samples show significantly higher photocatalytic activity than ZnO. CZO-4 has the highest photocatalytic performance. It can be found that the degradation rate of RhB first increases and then decreases with the increase of Ce doping. The photocatalytic degradation rate follows the order from highest to lowest activity: CZO-4 > CZO-3 > CZO-2 > CZO-1 > CZO-5 > ZnO.

The main reason Ce doping can act to enhance photocatalytic activity can possibly be attributed to the presence of cerium ions in the ZnO lattice, whose 4f energy levels serve as electron traps for photogenerated electrons. In this way, the recombination of photoinduced holes and electrons is suppressed, which accelerates the transfer of electrons to dissolve oxygen molecules and to produce more superoxide radicals to degrade probe dyes [9,22]. As a result, CZO-4 exhibits a superior catalytic performance to pristine ZnO. The change of morphology also contributes to the improvement of the photocatalytic performance of CZO, which will be further discussed in a later section. However, a further increase in the cerium concentration acts to lower the photocatalytic activity. The reason could be that the excess cerium ions alter the structure to serve as the recombination center [26].

### The orthogonal experiments

The design and results of the photocatalytic experiments for the orthogonal tests are listed in Table 1.

**Table 1:** Design and results of the orthogonal experiments.

No.	*A*	*B*	*C*	*D*	Degradation rate/%

1	1	1	1	1	77.14
2	1	2	2	2	26.67
3	1	3	3	3	92.37
4	2	1	2	3	32.02
5	2	2	3	1	82.34
6	2	3	1	2	97.11
7	3	1	3	2	94.42
8	3	2	1	3	90.42
9	3	3	2	1	23.18

As shown in Table 2, pH was found to be the most influential factor. The optimum reaction conditions are as follows: RhB concentration of 10 mg/L, catalyst (ZnO) dosage of 0.7 g/L, pH 9, temperature of 50 °C. This result could serve as a beneficial reference for large-scale wastewater degradation.

**Table 2:** Range analysis of orthogonal experimental results.

	*A*	*B*	*C*	*D*

K_1_	65.39	67.86	88.22	60.89
K_2_	70.49	66.48	27.29	72.73
K_3_	69.34	70.89	89.71	71.6
R	5.09	4.41	62.42	11.84

### Recycling experiments

**Figure 2:**
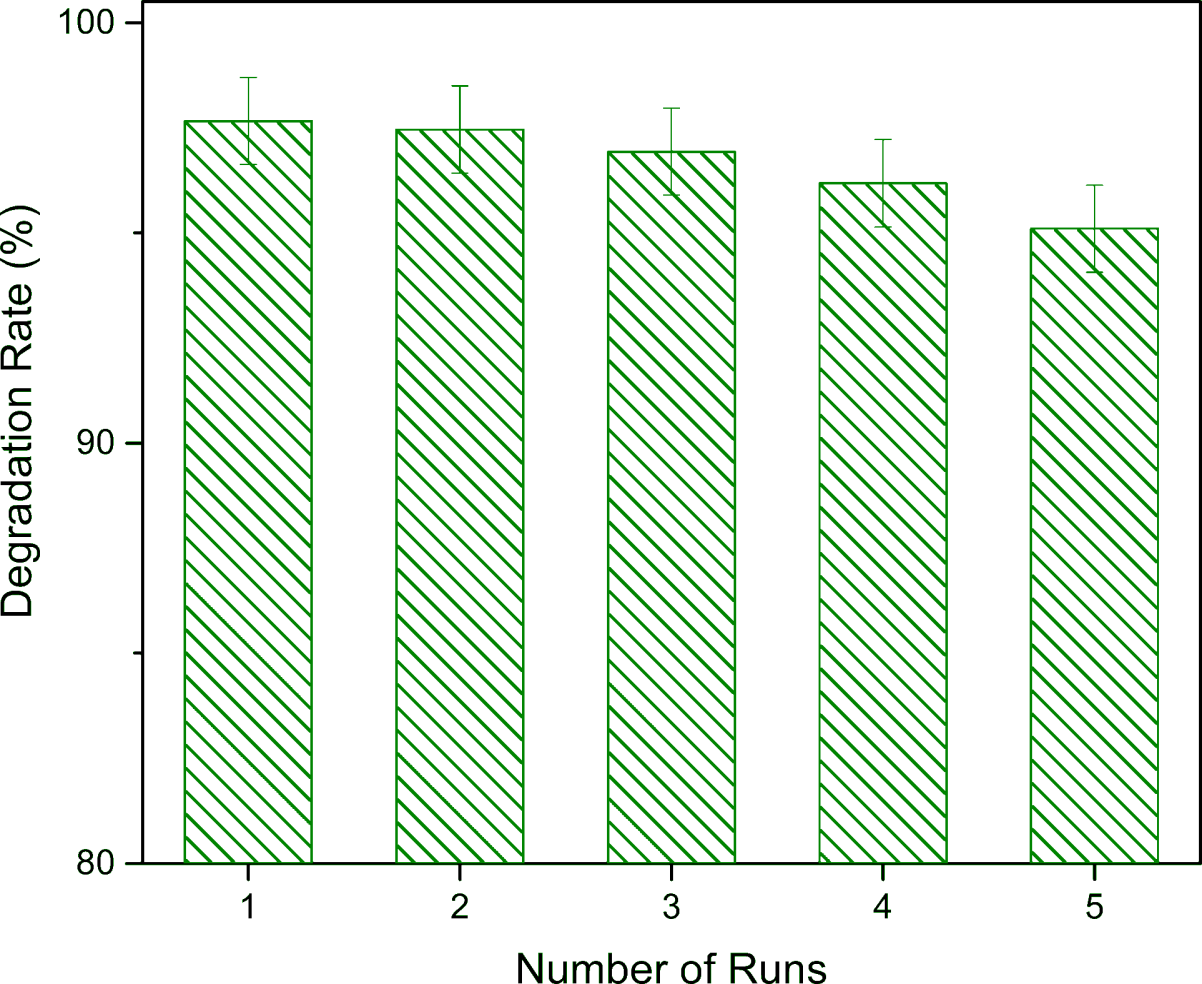
The cycle usage of the CZO-4 photocatalyst under optimized conditions in 2 h.

To evaluate the stability and repeatability of CZO-4, recycling experiments were carried out at optimized conditions (concentration of RhB = 10 mg/L, catalyst dosage = 0.7 g/L, pH 9 and temperature = 50 °C). The photocatalysts were recycled after washing, centrifugation and vacuum drying at 75 °C for 1 h. The results given in Figure 2 demonstrate that after five runs, CZO-4 maintains its remarkable catalytic activity with only a minor decrease in the degradation rate observed from 97.66% to 95.1%, indicating its excellent reusability [27]. Also, we found that RhB can be almost completely degraded with a degradation rate of 99.63% in about 3 h.

### Characterization

**Figure 3:**
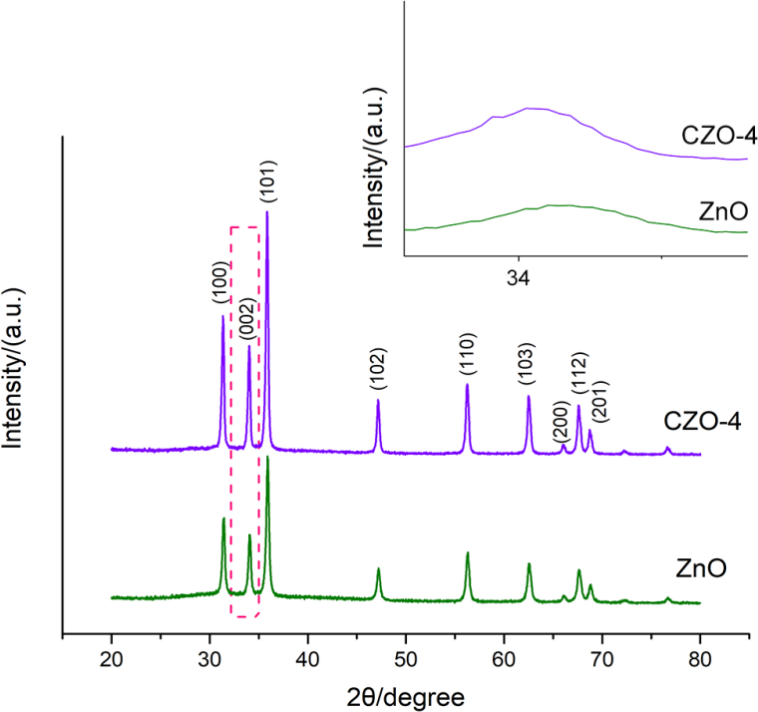
XRD patterns of ZnO and CZO-4 (3% Ce doping).

The XRD patterns of pure ZnO and CZO-4 are given in Figure 3. It can be found that the sharp peaks of ZnO, which indicates a high degree of crystallinity, are located at 2θ values of 31.5, 34.05, 35.9, 47.2, 56.3, 62.6, 66.08, 67.64, 68.84 finely corresponding to the (100), (002), (101), (102), (110), (103), (200), (112) and (201) crystal planes. These observations are consistent to those of wurtzite hexagonal ZnO (JCPDS No. 36-1451). The characteristic peaks of CZO-4 are located at 2θ values of 31.37, 34.01, 35.86, 47.17, 56.26, 62.48, 66.10, 67.58, 68.73, which indicates that the Ce-doped ZnO still retains the main diffraction peaks of ZnO, and no other additional crystalline impurities are generated. In this way, the cerium ions have been doped into the structure of ZnO [28–29]. Ahmad et al. [9] reported that Ce-doped ZnO has lower diffraction peaks after doping. However, our results showed that CZO-4 actually has higher diffraction peaks than pure ZnO, which means an increased crystalline quality and successful incorporation of cerium ions into the ZnO lattice [30–32]. The XRD patterns were analyzed in detail in the range of 33–35° as shown in Figure 3. It can be found that the diffraction pattern of CZO-4 shifts slightly towards lower angles compared to that of ZnO. Considering that the radius of the Ce^3+^ ion (0.102 nm) is much larger than that of Zn^2+^ (0.074 nm), such a shift might result from the fact that the cerium ions have been doped into the ZnO lattice and substituted into the Zn ion sites [9,11].

**Figure 4:**
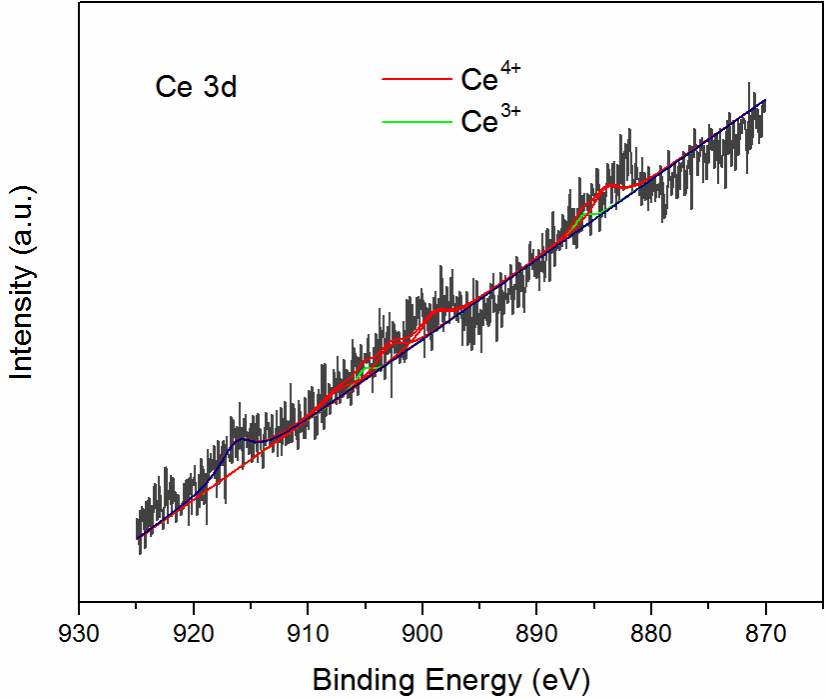
Ce 3d XPS of the catalyst CZO-4.

XPS spectra of Ce 3d were collected in order to determine the Ce state in CZO-4. All the elements were corrected with C 1s at 284.8 eV. Due to the low percentage of Ce in this catalyst, its signal to noise was low, too. The results are shown in Figure 4. We calculated the Ce state using curve-fitting based on the literature [33]. The results revealed that the percentage of R_Ce_^3+^ (Ce^3+^/(Ce^3+^ + Ce^4+^)) and R_Ce_^4+^ (Ce^4+^/(Ce^3+^ + Ce^4+^)) was 26.4% and 73.6%, respectively.

**Figure 5:**
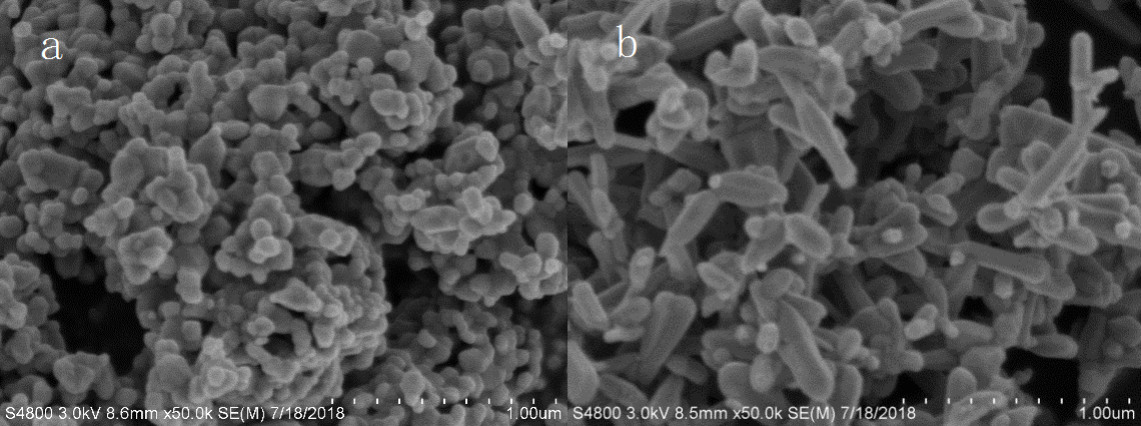
SEM micrographs of (a) ZnO and (b) CZO-4 (3% Ce doping).

To examine the surface morphology, SEM was carried out. Figure 5 shows SEM images of ZnO and CZO-4. Obvious differences can be observed between the undoped and cerium-doped ZnO. As demonstrated in Figure 5a, pristine ZnO has a spherical microstructure, while CZO-4 reveals a rod-like microstructure as shown in Figure 5b. It was also reported by Zhou et al. [34] that the as-synthesized Ce-doped ZnO produced via the hydrothermal method exhibits a well-distributed rod-like morphology, whereas the pristine ZnO consists of irregular shaped structures. The distinct difference in the surface morphology further confirms the successful cerium doping.

In this study, unlike the irregular and dense structure of ZnO, we can discern that CZO-4 has a rod-like structure, which makes it more distributed, resulting in the hollow microstructure and the outstanding photocatalytic activity. With respect to the mechanism of morphology change, it can be speculated that the doping of cerium ions affects the formation of ZIF-8. More specifically, it undergoes three main stages: gel formation, nucleation and crystallization [23]. According to Mahmoud’s [35] report, the Ce element which is doped into the lattice of Zn seeds may lower the surface energy, thus leading to a new morphology. Labhane et al. [36] reported a series of Ce-doped ZnO with different molar ratio by using the co-precipitation method. The SEM images of their products demonstrated that as the molar ratio is increased, the spherical nanoparticles changed to irregular and elongated shapes, which is similar to our results. In their study, Ce-doped ZnO (Ce/Zn 5%) has an average decolorization rate of 361.57 µg/h towards methylene blue, far outweighing the rate of 142.9 µg/h using ZnO.

Also, the concentration of Zn and Ce in the CZO-4 was analyzed by ICP-MS and the results are shown in Table 3. From the results in Table 3, we calculated the practical concentration of Zn and Ce in CZO-4. Zn% (practical) = (6.172 mg/L)/(8 mg/L) × 100% = 77.15%. Ce% (practical) = (0.065 mg/L)/(8 mg/L) × 100% = 0.8125%. These results indicate that the cerium ions had been successively doped into the ZnO lattice, accounting for the change of surface morphology and better photocatalytic activity.

**Table 3:** ICP-MS data of Zn and Ce in the CZO-4 sample. Standard deviation (STD), relative standard deviation (RSD).

	Mean corrected	Calibrated		Sample		

Analyte	Intensity	Concentration	STD	Concentration	STD	RSD
Zn 206.200	288353.3	6.172 mg/L	0.0288	6.172 mg/L	0.0288	0.47%
Ce 413.764	7642.2	0.065 mg/L	0.0014	0.065 mg/L	0.0014	2.09%

### Surface field

**Figure 6:**
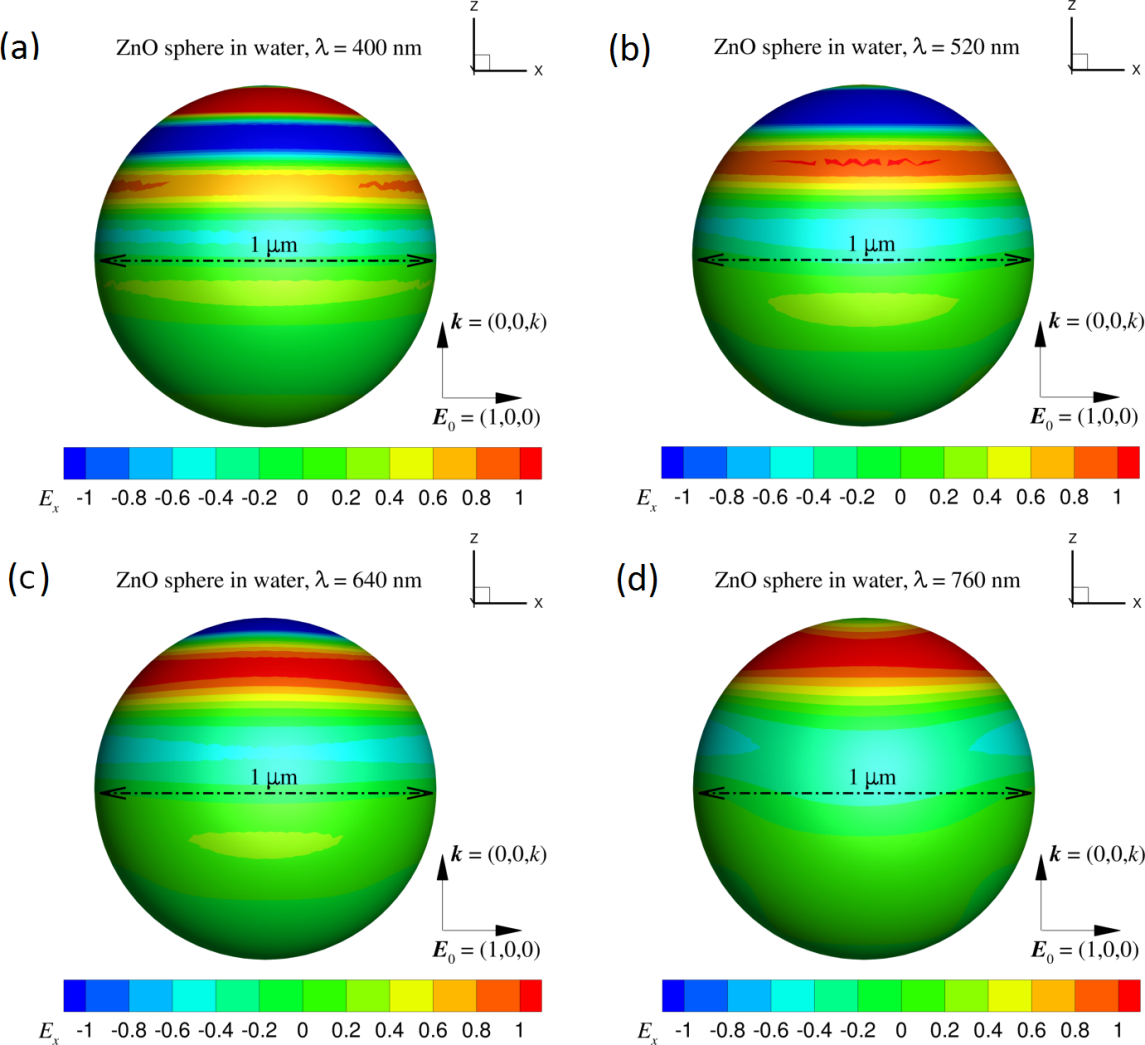
The total electric field on the surface of a ZnO sphere with diameter of 1 µm due to an incident electric field *E*^inc^ = (1,0,0) exp(*ikz*) where λ = 400 to 760 nm. The magnitude of the field, *E*_x_, is given by the color scale.

**Figure 7:**
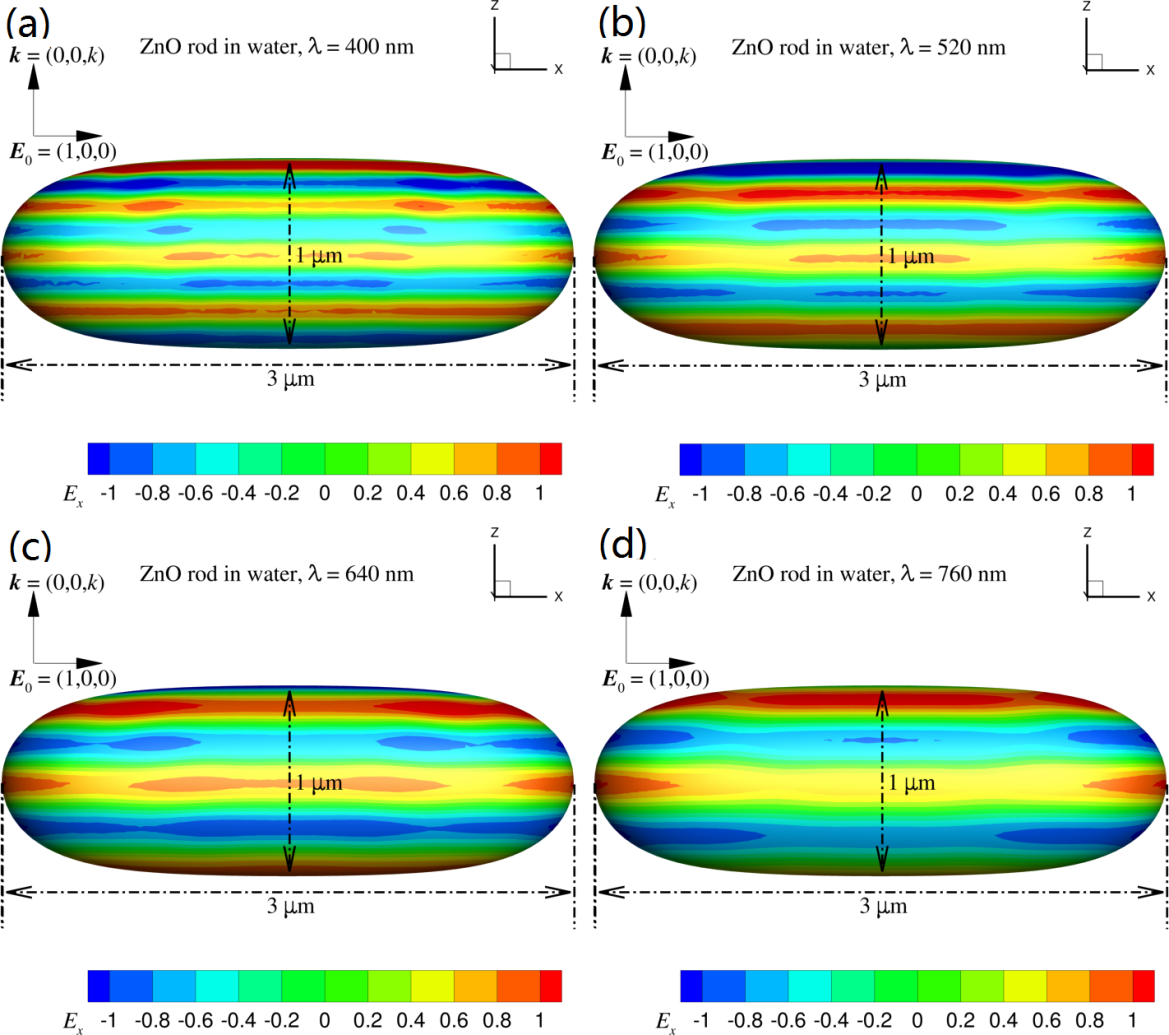
The total field on the surface of a ZnO rod with length of 3 µm and width of 1 µm due to an incident electric field *E*^inc^ = (1,0,0) exp(*ikz*) with λ = 400 to 760 nm. The magnitude of the field, *E_x_*, is given by the color scale.

The unique shape and porous microstructure are believed to have a great impact on the photocatalytic activity of catalysts by concentrating the reactant and scattering the light (high-performance rods). As an initial attempt to verify this conclusion, we ran a few numerical tests on how the particle shape can alter the electromagnetic field on the particle surface by using the newly developed robust field-only surface integral method [37–38]. For simplicity, and also without losing generality, we considered a single ZnO particle submerged in water with refractive index *n* = 1.33. The sunlight stimulator was represented by a time-harmonic incident plane wave with wavelength λ = 400–760 nm. The incident wave was set to propagate along the *z*-direction and polarize along the *x*-direction (the long-axis of the ZnO rod). According to Figure 3, we chose a ZnO sphere with diameter of 1 µm and a ZnO rod with length of 3 µm and width of 1 µm for comparison (refractive index was given by Querry [39]). From the results in Figure 6 and Figure 7, we can see that the electric field along the propagation direction of the incident field on the ZnO sphere is different to that on the ZnO rod. The obvious difference is that the dipole-like field on the ZnO sphere is always kept on the top half of the particle with respect to the wave propagation direction which decreases as the wavelength increases. On the surface of the ZnO rod, the dipole-like field is expanded to cover the entire surface, which is maintained over the entire wavelength range considered. This feature of the well-behaved dipole-like field on the rod surface could contribute to the improvement of the photocatalytic activity.

## Experimental

ZIF-8 was firstly prepared as a precursor, then ZnO and a series of Ce-doped ZnO were synthesized by a one-step pyrolysis method. The photocatalytic activity was evaluated by the degradation rate of RhB using a sunlight stimulator. The changes in crystal structure, Ce element state, morphology and chemical composition were characterized by XRD, XPS, SEM and ICP, respectively. Furthermore, to supply a helpful reference for large-scale dye degradation, the optimum photocatalytic conditions of undoped and doped catalysts were also screened.

### Sample preparation

#### Materials and instruments

In this study, zinc acetate dihydrate was purchased from Wuxi ZhanWang Chemical Reagent Co., LTD. 2-methylimidazole was provided by Shanghai Bide Pharmaceutical Technology Co., LTD. All other reagents used were purchased from Sinopharm Group Chemical Reagent Co., LTD.

A Newport 94023A (USA) device was used as the sunlight stimulator. The UV–vis spectroscopy and centrifugal operation were carried out on DU730 and AllegraTM X-22R devices (BeckMan Coulter). XRD, XPS and SEM were performed on D8 Advance (Bruker, Germany), Thermo Escalab 250 XI (PHI-5000 Versaprobe, UK) and S-4800 (Hitachi, Japan) devices, respectively. An in-house FORTRAN code was developed to implement the field-only surface integral method used to calculate the electric field on the particle surface.

#### Synthesis of ZnO

Zn(OAc)_2_·2H_2_O (4.39 g, 0.02 mol) and 2-methylimidazole (6.16 g, 0.075 mol) were dissolved in 150 mL of methanol (MeOH). Then the solution of 2-methylimidazole was poured into the solution of Zn(OAc)_2_·2H_2_O. After stirring at room temperature for 12 h, the white precipitate (ZIF-8) was centrifuged and washed thoroughly with MeOH and dried at 60 °C for 1 h [40]. After calcination at 500 °C for 3 h, the zinc oxide micro–nanomaterial was obtained.

#### Synthesis of CZO

For the synthesis of CZO, cerium nitrate hexahydrate (Ce(NO_3_)_3_·6H_2_O) was selected as the doping material during the stirring process. The operational steps were the same as the synthesis of ZnO. Eventually, we obtained five groups of CZO samples with different concentrations. For simplicity, samples with molar ratio of 0.5%, 1%, 2%, 3%, and 4% were named as CZO-1, CZO-2, CZO-3, CZO-4, and CZO-5, respectively.

### Photocatalytic activity measurements

The photocatalytic activity was evaluated based on the degradation of RhB using a sunlight stimulator. First, 4 mg of the photocatalyst was added to the RhB aqueous solution (8 mL) with a concentration of 10 mg/L in numbered test tubes. The suspension was stirred for 30 min to reach the adsorption/desorption equilibration in darkness. Following this, the photocatalytic reaction was started by turning on the solar simulator. 1 mL of the suspension was sampled and centrifuged before this process began. The supernatant was diluted with an equal amount of deionized water, then taken out for the measurement of absorbance (*A*_0_) at 554 nm by UV–vis spectroscopy. Every 20 min the sampling process was repeated, and the absorbance was determined. The degradation rate (DR) was calculated using the equation: DR = [(*A*_0_ − *A*)/*A*_0_] × 100%.

### Orthogonal design

Referring to the orthogonal table of L9 (3^4^), nine experiments with different concentrations of RhB (*A*, mg/L), catalyst amount (*B*, mg/mL), pH value (*C*), and temperature (*D*, °C) were carried out. Experimental factors were summarized in Table 4. The degradation rate of RhB was taken as the response value.

**Table 4:** Table of experimental factors for the orthogonal table of L9 (34). Nine experiments with different concentrations of RhB (*A*, mg/L), catalyst amount (*B*, mg/mL), pH value (*C*), and temperature (*D*, °C) were performed.

Level	*A*, mg/L	*B*, mg/L	*C*	*D*/°C

1	5	0.3	5.65^a^	40
2	10	0.5	4	50
3	15	0.7	9	60

^a^Original pH.

Taking number 2 in Table 1 as an example, the typical experimental steps are as follows: 4 mg CZO was added to 8 mL RhB solution (0.5 mg/mL) with a moderate amount of NaOH solution and acetic acid solution to maintain pH 4 in a test tube. The suspension was stirred in darkness for 30 min before the initial absorbance was measured. The degradation rate was calculated after 2 h of exposure to sunlight. The design and results are listed in Table 1.

### Characterization of photocatalysts

The XRD patterns of ZnO and CZO-4 were recorded using an X-ray micro-diffractometer with Cu Kα radiation (λ = 0.15406 nm). The samples were ground into a fine powder in an agate mortar before the tests. To figure out the state of the Ce element, 30 mg of photocatalyst was sampled for the XPS analysis on an Axis Ultra DLD Kratos AXIS SUPRA system, using Al Kα as a monochromatic radiation source. The specific surface morphology was observed by an SEM with a secondary electron resolution of 1.0 nm (15 kV) and 2.0 nm (1 kV) with a magnification of ×30 to ×800000. SEM images of the samples at different magnifications were obtained. Clarified aqueous solutions of ZnO and CZO-4 (8 ppm) were used to measure the specific chemical compositions.

## Conclusion

In this study, we synthesized spherical ZnO and a series of rod-like Ce/ZnO catalysts via high temperature pyrolysis to investigate their photocatalytic performance using RhB as a model pollutant. The photocatalytic activity of Ce/ZnO with different doping concentrations was evaluated using a sunlight simulator. The factors, including RhB concentration, catalyst amount, temperature and pH value, on the photocatalytic activity were optimized by an orthogonal design method. The maximum RhB degradation of 97.66% was observed under optimum conditions. The XRD, XPS, SEM and ICP characteristic data revealed that the Ce-doped ZnO with special rod-like shape can remarkably improve the photocatalytic degradation against RhB. To explain this impressive photocatalytic activity, the robust field-only surface integral method was employed firstly to explore the possible mechanism of this rod-like Ce-doped ZnO photocatalyst. This computational result revealed that the specific surface area of this rod-like photocatalyst provides conditions for the enhancement of catalytic performance.
